# Acute Urinary Retention in a Patient With Heart Failure and Benign Prostatic Hyperplasia: A Case Report

**DOI:** 10.7759/cureus.69848

**Published:** 2024-09-21

**Authors:** Abdulrahman Tabrah

**Affiliations:** 1 Internal Medicine, Al-Furat General Hospital, Baghdad, IRQ

**Keywords:** "old age", bph, transient sudden hypotension, heart failure, acute urine retention

## Abstract

This case report discusses the presentation, management, and complications of acute urinary retention in a 70-year-old male with a history of heart failure, hypertension, diabetes, and benign prostatic hyperplasia (BPH). The case highlights the occurrence of post-catheterization hypotension, a known but poorly understood phenomenon, and underscores the need for a multidisciplinary approach in managing patients with complex medical conditions.

## Introduction

This case report dives into the presentation, management, and complications of acute urinary retention (AUR) in a 70-year-old man dealing with heart failure, high blood pressure, diabetes, and benign prostatic hyperplasia (BPH). His medications are as follows: furosemide tab 40 mg once daily po, captopril tab 25 mg twice daily po, amlodipine 10 mg once daily po, metformin 500 mg twice daily po, carvedilol 6.25 twice daily po, and tamsulosin tab 0.4 mg once daily po. It sheds light on the not-so-well-understood occurrence of post-catheterization hypotension, giving different possibilities of the cause and stressing the importance of a multidisciplinary approach when managing patients with such a complex web of health problems [1].

## Case presentation

Patient information

The patient, a 70-year-old male, came in with a pretty heavy list of health issues: heart failure managed with diuretics, high blood pressure on calcium channel blockers, and type 2 diabetes controlled with oral meds. He had a recurring history of AUR due to BPH and was on alpha-blockers, waiting for surgery [1].

Clinical presentation

He showed up in the emergency department with AUR for 12 hours, his fourth attack during the last two months. He was awake, alert, and aware, but an abdominal exam showed a soft abdomen but a distended bladder, meaning it was quite full. Listening to his lungs revealed some fine crackles at the base both likely tied to his heart failure. His blood pressure was up at 180/100 mmHg, with a heart rate of 95 and an oxygen saturation of 95% on room air. Despite these issues, his initial lab tests, checking kidneys, electrolytes, and full blood, looked normal (see Table 1). Ultrasound at the bedside confirmed what we suspected: a significantly distended bladder.

**Table 1:** Laboratory findings

Test	Patient result	Normal range
Serum creatinine	1 mg/dL	0.6-1.2 mg/dL
Urea	6.5 mmol/L	2.5-7.5 mmol/L
Hemoglobin	14 g/dL	13.8-17.2 g/dL (men)
White blood cells	9,000/µL	4,000-11,000/µL
Platelets	164,000/µL	150,000-450,000/µL
Sodium	136 mmol/L	135-145 mmol/L
Potassium	4.0 mmol/L	3.5-5.0 mmol/L
Chloride	105 mmol/L	98-106 mmol/L
Bicarbonate	24 mmol/L	22-26 mmol/L

**Figure 1:**
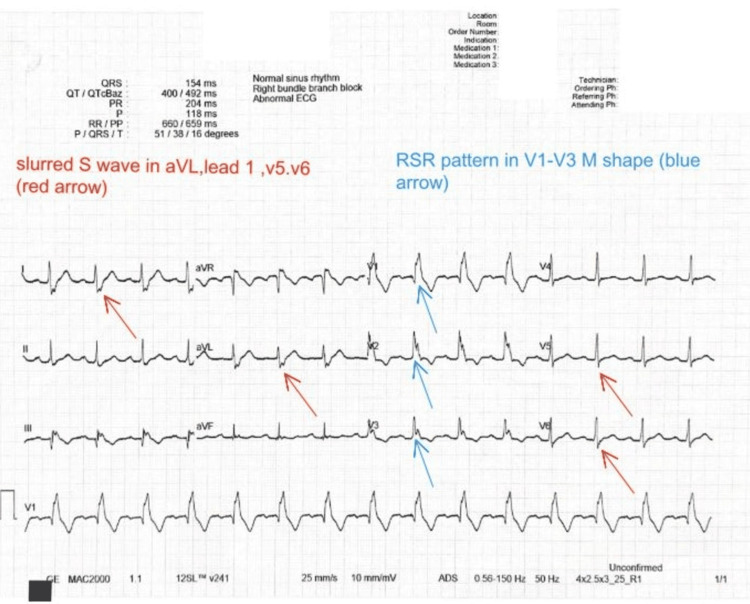
ECG The patient's ECG showing RBBB features: red arrows showing a slurred S wave in lateral leads (aVL and leads 1, V5, and V6), blue arrows showing an RSR M-shaped pattern in V1-V3, and QRS complex wider than 120 ms ECG: electrocardiogram; RBBB: right bundle branch block

Once he was admitted, two IV lines were put in, and an electrocardiogram (ECG) (Figure 1) was done to rule out any acute heart issues; it showed a right bundle branch block, an old finding noted in his records, with no new cardiac events. We used a Foley catheter to drain his bladder, and about 2000 mL of urine came out. But then things took a turn; he suddenly got pale, started sweating, and felt dizzy, his blood pressure dropped sharply to 90/70 mmHg, and his pulse rate (PR) became 110 with normal SpO2. We had to act fast: we laid him flat, gave him fluids quickly, and put him in a central line to keep a close eye on his blood pressure.

Outcome and follow-up

Thankfully, after the fluids, his blood pressure picked back up. We kept monitoring him closely and were on the lookout for post-obstructive diuresis, a situation that could mess with his heart failure even more. We made a team of specialists, urology, cardiology, and endocrinology, to make sure he got the best care possible.

## Discussion

AUR can be a real nightmare, especially when it pops up in patients who already have a lot going on medically. This case of a 70-year-old man with heart failure, BPH, high blood pressure, and diabetes shows just how tricky things can get. What stands out here is post-catheterization hypotension, which is a drop in blood pressure after draining the bladder. It needs careful management, particularly in people with heart issues.

We know that AUR is common in men with BPH and often needs fast treatment to avoid other problems [1]. This patient's repeated episodes of AUR, along with his ongoing therapies for heart failure and high blood pressure, highlight how vital it is to have a team of experts working together. Standard treatment for AUR usually involves draining the bladder with a catheter [2], but in this case, we saw how quickly things can go south with hypotension afterward. It's thought that this might be due to rapid changes in fluid levels and blood flow or the polypharmacy the patient is taking, but honestly, we still have a lot to learn about this [3].

Managing AUR in patients with other health conditions means being super careful about the risk of post-obstructive diuresis, which can worsen heart failure symptoms and lead to more drops in blood pressure, like what happened here [1]. Handling the sudden low blood pressure with symptoms like pallor, sweating, and dizziness required quick fluid replacement and keeping a close watch on his vitals [2]. This approach lines up with best practices for these kinds of complications in patients who have a lot of health issues going on [3].

Getting a multidisciplinary team on board, including urology, cardiology, and endocrinology specialists, was key in this case. Their combined efforts were crucial to navigating the complex nature of AUR in a patient with so many other problems [1,2]. The steps we took, fluid resuscitation, central line placement, and constant monitoring, represent the gold standard for dealing with the hemodynamic challenges that come with AUR and its complications [3].

## Conclusions

This case shows just how complicated it can be to manage AUR in someone juggling multiple health issues, like heart failure and BPH. The sudden drop in blood pressure after catheterization drives home the need for careful monitoring and quick responses. A team approach in the expertise of urology, cardiology, and endocrinology is not just helpful; it's necessary for managing such cases effectively and making sure the patient gets the best possible outcome. We need more research to better understand the causes of post-catheterization hypotension (maybe it is due to polypharmacy or vasovagal effect or a hidden reason that will be revealed with continuous research) and how to handle it, especially in patients with complicated medical histories, so we can keep improving our treatment approaches and care.
